# The social support networks of elderly people in Slovenia during the Covid-19 pandemic

**DOI:** 10.1371/journal.pone.0247993

**Published:** 2021-03-03

**Authors:** Marjan Cugmas, Anuška Ferligoj, Tina Kogovšek, Zenel Batagelj

**Affiliations:** 1 Faculty of Social Sciences, University of Ljubljana, Ljubljana, Slovenia; 2 National Research University Higher School of Economics, Moscow, Russia; 3 Faculty of Arts, University of Ljubljana, Ljubljana, Slovenia; 4 Valicon, Ljubljana, Slovenia; Texas State University, UNITED STATES

## Abstract

Population ageing requires society to adjust by ensuring additional types of services and assistance for elderly people. These may be provided by either organized services and sources of informal social support. The latter are especially important since a lack of social support is associated with a lower level of psychological and physical well-being. During the Covid-19 pandemic, social support for the elderly has proven to be even more crucial, also due to physical distancing. Therefore, this study aims to identify and describe the various types of personal social support networks available to the elderly population during the pandemic. To this end, a survey of Slovenians older than 64 years was conducted from April 25 to May 4, 2020 on a probability web-panel-based sample (n = 605). The ego networks were clustered by a hierarchical clustering approach for symbolic data. Clustering was performed for different types of social support (socializing, instrumental support, emotional support) and different characteristics of the social support networks (i.e., type of relationship, number of contacts, geographical distance). The results show that most of the elderly population in Slovenia has a satisfactory social support network, while the share of those without any (accessible) source of social support is significant. The results are particularly valuable for sustainable care policy planning, crisis intervention planning as well as any future waves of the coronavirus.

## Introduction

The Covid-19 pandemic (SARS-CoV-2) has affected masses of people around the world, with some being disproportionally exposed to the associated risk due to their age, employment status, financial status, illness or other factors [1, 2]. One such group is the elderly, who are not only vulnerable by being at greater risk of death if infected [3–5], but also because those whose only social contacts are outside the home are more likely to lack social support [6] due to the preventive measures imposed to reduce the coronavirus’ spread (e.g., physical distancing and self-isolation).

Social support is a multidimensional concept that may be defined as “the aid—the supply of tangible or intangible resources—individuals gain from their network members” [7]. In general, studies show that actual and perceived social support positively affects (mental) health [7]. However, when providers of social support are unreliable or when receiving social support is very demanding or includes conflicts, it may produce negative effects for mental health [8–10]. Nevertheless, social support usually has a positive impact on health, especially for preventing depression and anxiety [7, 11], whereas the absence of social support (i.e., perceived social isolation and loneliness) increases the risk of mental disorders like depression and anxiety [12].

The latter—depression and anxiety—are common psychological reactions to the Covid-19 pandemic [13, 14], therefore making social support vital for maintaining health during this period. A German study reveals that perceived social support is related to lower levels of anxiety, depressive and sleeping disorders during the coronavirus pandemic [13], while a Chinese study shows greater psychological distress among adult Chinese with less social support during the Covid-19 pandemic than those who have more social support [15].

As mentioned, the increased levels of these symptoms might be related to the social distancing and self-isolation requirements. A study in the United States found higher levels of anxiety, financial worry, and loneliness among those who live in counties with a stay-at-home order compared to other counties [16].

Elderly people, especially those with little social support, living alone, and already suffering from a mental disorder [17, 18] or another chronic disease, are particularly vulnerable. A recent study in Spain of a sample of the elderly with a mild cognitive impairment and mild dementia during a coronavirus-related quarantine [19] reveals that those living alone reported more prominent adverse psychological effects and trouble sleeping than others. Studies before the time of Covid-19 showed that a bigger number of network ties, more contacts with similar people, and more intensive contacts with unknown people is associated with fewer illnesses and diseases among the elderly [20]. The life quality of elderly people with age-related hearing loss was connected to social support while the number of other associated illnesses and diseases—the quality of life was higher where the level of social support was perceived to be higher, and lower with a bigger number of related illnesses and diseases [21].

A Polish study [22] among the elderly also pointed to the effect of the environment: in urban environments, social network and social participation were linked with a positive estimation of one’s health. In rural environments, a positive self-estimation of health was also influenced by social support and education, while a lower self-estimation of health was influenced by a greater level of loneliness.

The above studies demonstrate that the characteristics of elderly people’s personal social networks are connected to their physical and psychological health. However, since social support is a multidimensional construct and elderly people are an incredibly heterogeneous population (the ageing process itself is highly diverse and context-dependent) [23], much effort has been made to identify the different types of personal social support networks among them, given that some are more vulnerable than others due to the type of social support they have available. Wenger [24], for example, identifies five support network types of the elderly population:

1. **Family-dependent support network**. Elderly people in this network type share the household with their child (or the child lives close by), who mostly takes care of their needs. Friends and relatives also help in a peripheral role. The networks are small while the elderly are often widowed and older (aged 80+).
2. **Locally integrated support network**. This network type is typified by close relationships with friends and neighbors, and active community involvement. The networks are relatively large, and the elderly people are not as old (aged 65–74).
3. **Self-contained support network**. In this network type, is typical relationship is with a relative who might live further away. As there are generally no children, assistance mainly comes from the neighbors, which considerably isolates the household. The networks are small and the person lives alone.
4. **Wider community-focused support network**. The characteristics of this type are active relationships with distant relatives (usually children), close friends, and neighbors. The networks are relatively large.
5. **Private restricted support network**. In these networks one finds independent married couples or extremely isolated elderly people. The networks are small, relatives do not live nearby, there is no contact with the neighbors, but there might be a few close friends living in the vicinity.

The most vulnerable elderly people are those with the third and fifth types of social support network because they do not have enough sources of assistance in the event of illness or a greater need for social support and may require institutional care. The risk is higher in countries where institutional aid is not readily accessible (e.g., long waiting lists to enter retirement homes) and where access to home care varies substantially between local communities—in terms of both adequate staff capacities and municipal subsidies.

Following Wenger’s typology [24], Hlebec [25] performed a typology of the Slovenian elderly population in 2000 and detected six types of elderly networks:

**First network type**. Individuals in this network type are the wealthiest and most educated. The social support network is primarily composed of friends (less so of relatives), while there are almost no neighbors. It is geographically the most widespread and relatively large.
**Second network type**. The social support network is chiefly composed of relatives, marked by their geographical closeness; many married couples.
**Third network type**. The elderly with this network type are mostly women living alone. Many are widowed with generally low education and income levels. Their social support networks are relatively large, predominately family-dependent, some neighbors. Network members live close by.
**Fourth network type**. Those with this type of social support network are married with a relatively low level of education, but a higher income. Their households are typically extended (two generations living together) and located in a rural environment. Their social support networks are relatively large and their primary source of support is their relatives.
**Fifth network type**. This network type is similar to the third network type. Social support networks are smaller and oriented more to friends and neighbors than to family.
**Sixth network type**. The elderly with this network type are mostly less educated and poorer women. This type of social support network is strongly determined by children and the absence of other sources of support.

To summarize, the identified networks are mainly family-dependent, one type may be compared with Wenger’s [24] Wider community-focused support network type and another with the Locally integrated support network type. The sixth type could also be called the Private restricted support network type. According to Hlebec [25], elderly people with the latter support network type might be especially vulnerable as others have relatively well-equipped networks of social support with network members who live close to them.

### Objectives of the research

While elderly people are generally more vulnerable and need additional care and services in both coronavirus and non-coronavirus times, one must consider that levels of vulnerability vary among different groups of the elderly, also regarding the type of their social support networks, which can be helpful, deficient, or even harmful.

Accordingly, this paper aims to identify general characteristics of the social support networks of elderly people in Slovenia during the Covid-19 pandemic and to identify various social support network types. It is hypothesized that the identified network types will be consistent with the network types theoretically proposed by Wenger [24].

Moreover, the paper aims to discuss which types of social support networks might be the least efficient for dealing with issues related to the coronavirus pandemic and how many elderly people possess specific types of social support networks.

Knowledge of types of elderly people’s social support networks can guide practitioners while designing appropriate interventions and combinations of care services [3].

## The Covid-19 pandemic in Slovenia

**Fig 1:**
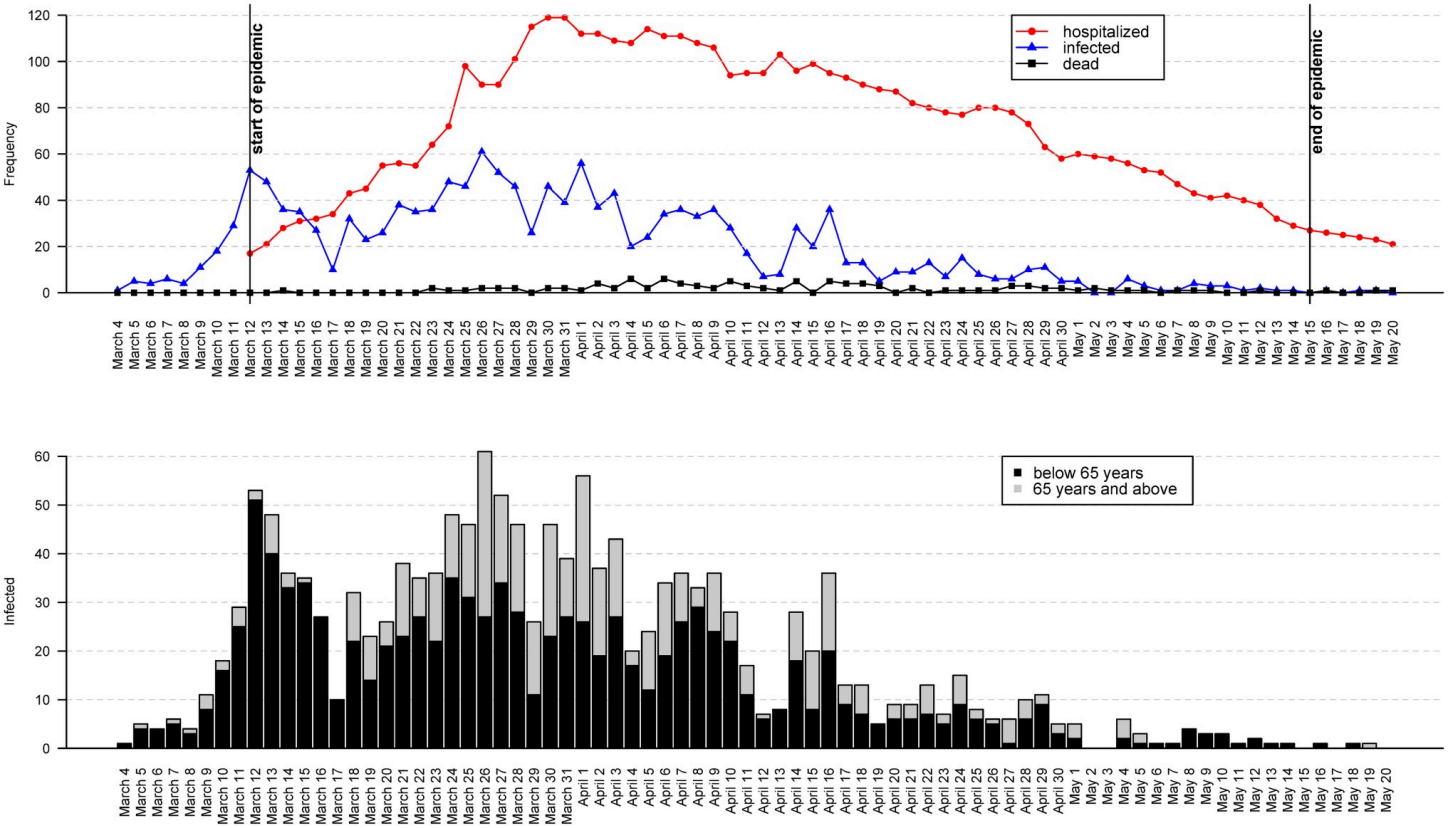
SARS-Cov-2 coronavirus in Slovenia. Upper panel: the number of infected, hospitalized, and dead persons in time. Lower panel: the number of infected persons in time by age groups.

The first person to be infected by the SARS-CoV-2 coronavirus in Slovenia was detected on March 4, 2020 [26]. The (first wave of the) epidemic in Slovenia officially started on March 12 and ended on May 15, 2020 [27, 28]. The number of infected persons had several peaks over time (Fig 1), more or less a consequence of the government’s measures to limit the coronavirus’ spread and of changing the testing regimes [29]. Soon after the epidemic had officially started, the Slovenian government imposed measures for general self-isolation, halted public transport services, and closed schools and universities. Only grocery stores and pharmacies remained open, while all other stores and catering establishments like cafes, bars, and restaurants were closed.

On March 18, the government introduced several other measures such as priority while visiting grocery shops between 8 AM and 10 AM for elderly people and other vulnerable groups (those with disabilities and pregnant women) [30], which entered into force on March 30 [31]. On the same day, the government also introduced a ban on leaving one’s home municipality (these are 212 small territorial areas with a median number of inhabitants of around 5,000; the smallest two municipalities have some 350 inhabitants) [32]. There were some exceptions, such as crossing over the boundaries of a municipality to visit family members in need of protection, assistance, support, or nursing care. Crossing between municipalities was also allowed for the purpose of visiting, e.g., grocery shops or pharmacies, if none existed in one’s own municipality. The reason for moving between municipalities had to be proved with appropriate documents.

For the elderly population and those with physical disabilities, the introduced measure about visiting grocery stores was insufficient because they were unable to complete their shopping by 10 AM. Especially vulnerable were individuals whose social supporters all came from other municipalities. Therefore, representatives of different governmental and non-governmental organizations suggested extending the opening hours for vulnerable groups [33], as happened on April 4 [34], allowing them to also visit grocery stores in the last hour of opening.

The number of infected persons started to fall in April while the number of deaths started to rise. Almost all of the deaths (98%) were among 65+ year-old persons, also because the virus had started to spread within retirement homes.

## Materials and methods

The egocentric network approach was used to measure social support in two steps: (i) **name generators** were used to obtain the list of people (alters) from which the respondent (ego) was receiving social support; then (ii) each ego provided several characteristics for each listed alter (**name interpreter**).

The construct of social support has at least four dimensions [11, 35, 36]: emotional support, instrumental support, socializing, and material support. The following relevant types of social support and corresponding name generators in the case of the Covid-19 pandemic were considered (see S1 Questionnaire or S2 Questionnaire): **informal socializing** (*“Who are the people you have been socializing with during the time of social isolation? This can be face to face or by phone, computer, tablet, and so on.”*), **emotional support** (*“To whom do you usually talk to these days about personal things that are important to you?”*), and **instrumental support** (*“In the coronavirus crisis, it is advisable not to leave your residence, e.g., to go shopping or to the pharmacy. To whom do you turn for this type of help?’*’).

Standard statistical methods and the most contemporary methods for analyzing complex data were applied to analyze the collected egocentric networks. The clustering of symbolic data approach [37] was used to obtain the typology of the egos’ networks. Compared to traditional clustering approaches where values (e.g., means, percentages) represent the characteristics of the egos’ networks, the symbolic data analysis enables a much more detailed analysis by considering the probability distributions of the characteristics of the egos’ networks. Therefore, in this study, the symbolic data for each ego are the probability distributions of the following measured characteristics of his or her social support network: (i) the distribution of the types of relationship of an ego to their alters; (ii) the distribution of the frequency of contacts with the alters; (iii) the distribution of the number of alters by type of social support; and (iv) the distribution of the geographical distances between an ego and their alters’ residences.

Besides the distributions listed above, the **number of alters**, (a variable) normalized to the interval [0, 1], was used in the clustering procedure. Clustering was performed using the clamix package [38] for the R programming language. In this package, Ward’s agglomerative method is implemented. The agglomeration can be represented by a dendrogram that allows the number of clusters to be determined.

This research was approved by the Workplace Ethics Committee (801-2020-045/JG) at the Faculty of Social Sciences, University of Ljubljana.

### Data

The data (in S1 Data) were collected by a web survey conducted from April 25 to May 4, 2020. The data collection occurred at JazVem (www.jazvem.si), a web portal maintained by the marketing and consulting company Valicon, and included people aged 65+. Written consent to participate in the study was obtained from the respondents.

Given that this is the probability sample of elderly adults from Slovenia who are capable and willing to participate in web surveys, there is no one from the retirement homes and no other people who are incapable of completing the survey for reasons like illness. It is estimated that between 35% (65+) and 47% (aged between 65 and 74 years old) of elderly people use the Internet in Slovenia. Among the 951 people invited to take part in the survey, 805 clicked on the link to the survey and 638 of them completed the entire survey. The response rate was relatively high (0.67). Of these, 605 respondents are considered in the analysis (33 respondents are excluded because they did not understand the name generators properly).

The sample is representative in terms of gender, age, and geographical region. Moreover, the final sample of 605 respondents does not differ significantly from the sample of 951 invited respondents regarding these characteristics.

## Results

**Fig 2:**
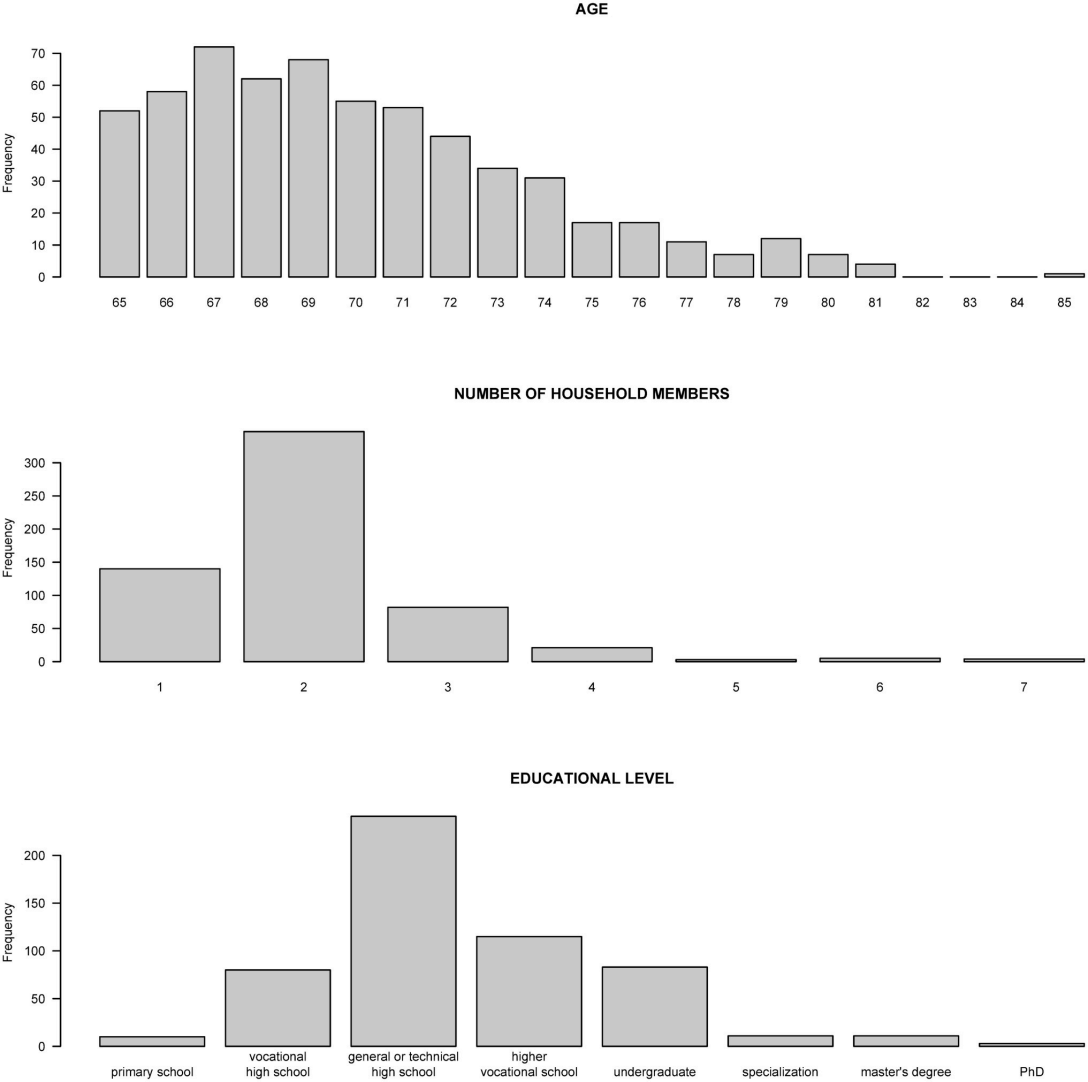
Distribution of age, number of household members, and education level.

In the sample, 48% of respondents come from an urban environment, 62% of respondents are married or have a partner, and the average age is 70 years (*min* = 65, *max* = 85, *sd* = 3.8) (Fig 2). As expected, 97% of the respondents are retired. The majority live with another person (58%) or live alone (23%) and most finished secondary school (51%) (Fig 2).

**Fig 3:**
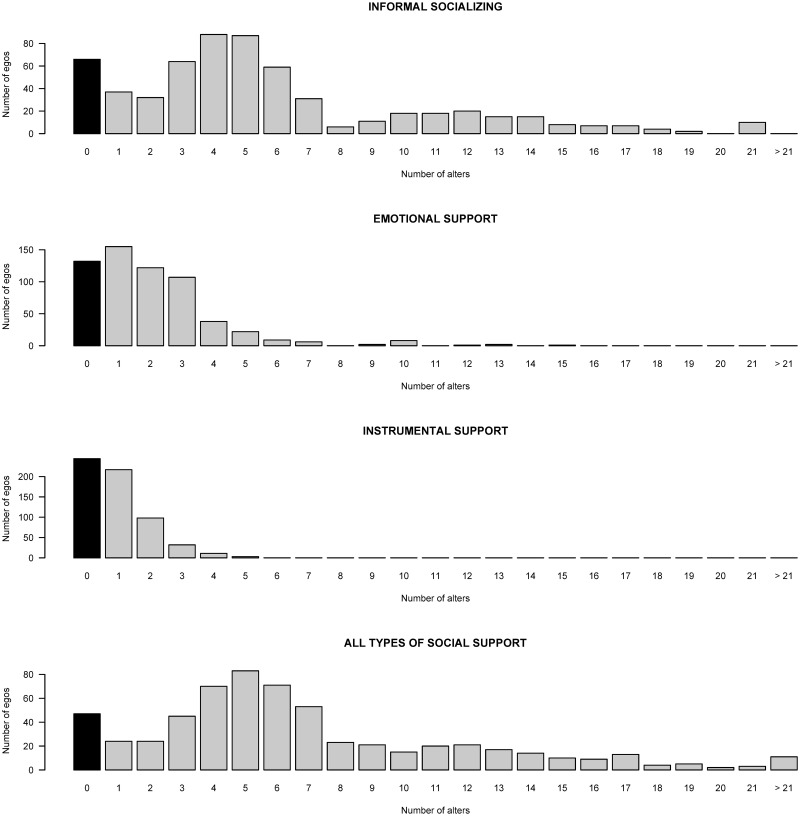
Distributions of the ego networks.

The egos listed a total of 4,163 alters. On average, an ego mentioned 6.9 alters. In Fig 3, the distributions of the number of alters for each of the three social support dimensions are given along with the distribution of all different alters of an ego.

**Fig 4:**
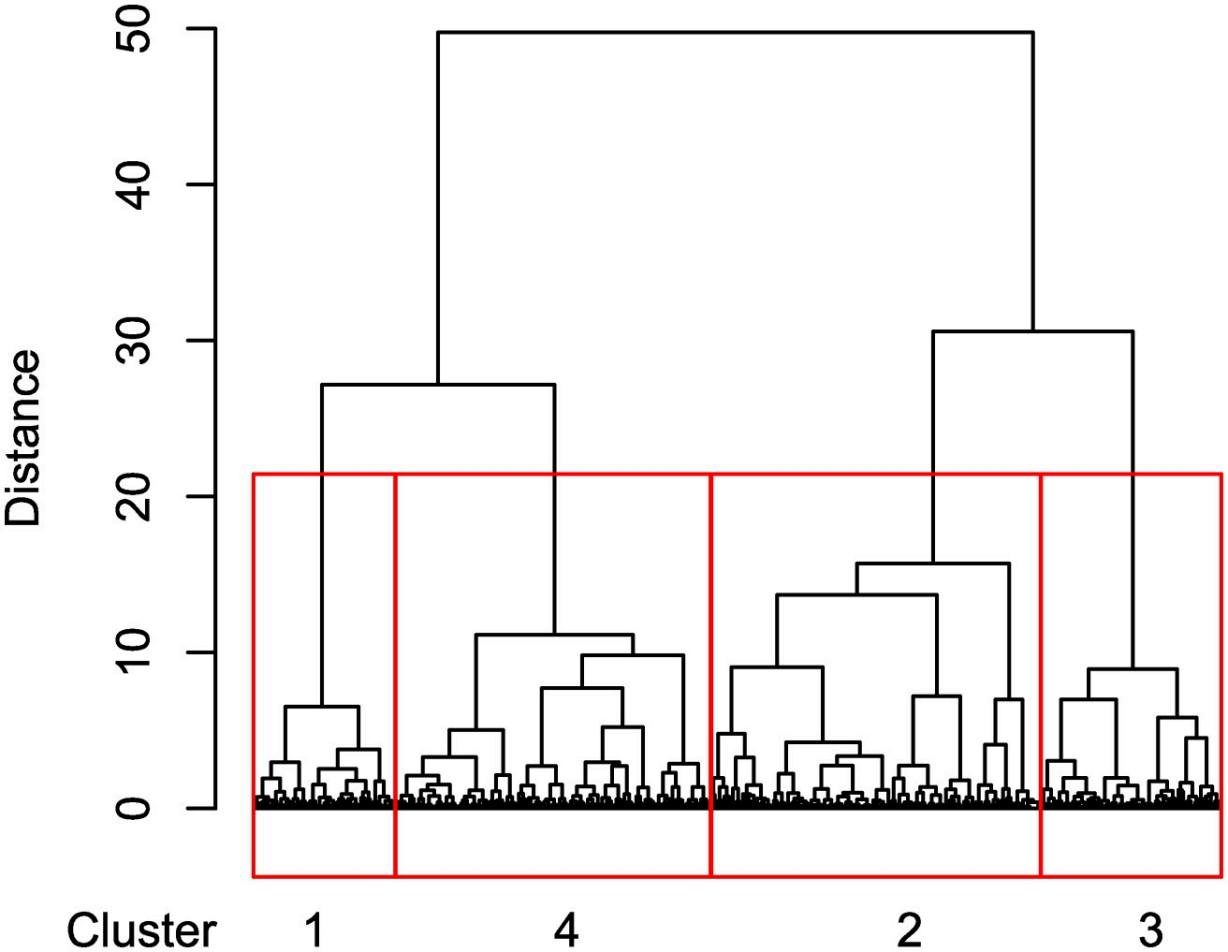
The dendrogram obtained by the hierarchical clustering approach of symbolic data.

The respondents have the highest number of alters for emotional support and the lowest for instrumental support. Overall, 47 egos did not mention any alter that provides him/her with social support. These elderly people are the most vulnerable. The second-most vulnerable elderly are those whose mentioned alters are all located in another municipality since it was forbidden to leave one’s municipality during the period in which strict measures were imposed. There are 27 of such respondents. We will consider these two groups of respondents in two separate clusters. For all the others, we applied the clustering of symbolic data approach. The obtained dendrogram of the hierarchical clustering of the symbolic data algorithm is presented in Fig 4.

The dendrogram reveals four distinct clusters. Their network characteristics are shown in Table 1 where the two previously mentioned clusters are added (Cluster 0 without any social support; Cluster 5 with all alters living in another municipality or abroad). The averages of alters by clusters are shown in Table 1 while descriptive statistics of the egos by clusters are shown in Table 2.

**Table 1:** The averages of alters by clusters.

CLUSTER	0	1	2	3	4	5	ALL
N	47	78	181	99	173	27	605
Total number of alters	0	10.0	5.5	6.1	9.5	4.3	6.9
Informal socializing	0	9.3	3.8	5.7	8.3	3.1	5.8
Emotional support	0	2.0	2.0	3.5	1.9	1.4	2.0
Instrumental support	0	0.9	1.2	1.3	0.8	0.6	0.9
**Type of relationship**							
Partner	0	0.7	0.6	0.6	0.5	0.1	0.5
Child	0	1.7	1.5	1.5	1.4	1.5	1.4
Grandchild	0	0.9	0.5	0.5	0.7	0.3	0.6
Other relatives	0	3.4	2.0	1.9	2.5	1.1	1.5
Friend	0	2.5	0.7	1.5	2.7	1.0	1.6
Neighbor	0	0.6	0.3	0.3	1.1	0.0	0.5
Other	0	1.0	0.4	0.4	1.4	0.6	0.7
**Number of roles**							
One role	0	8.2	2.2	2.7	8.3	3.6	5.3
Several roles	0	1.9	1.3	3.5	1.3	0.7	1.6
**Physical distance**							
Same household	0	1.0	1.4	0.7	0.8	0.0	0.9
Same municipality	0	1.9	2.6	2.4	5.5	0.0	3.0
Another municipality or abroad	0	7.1	1.5	3.0	3.3	4.3	3.0
**Frequency of contacts**							
Several times per day	0	0.9	1.8	0.8	0.8	0.2	1.0
Once per day	0	1.6	1.7	1.5	1.7	1.0	1.5
Several times per week	0	2.4	1.3	2.2	2.6	1.6	1.8
Once per week	0	3.1	0.6	1.0	2.6	0.9	1.5
Less often	0	2.0	0.2	0.7	1.8	0.6	1.0

**Table 2:** Descriptive statistics of egos by clusters.

CLUSTER	0	1	2	3	4	5	ALL
Mean age	69.6	69.5	69.7	70.1	70.6	70.3	70.0
Percentage of men	61.7	47.4	51.9	35.4	48.0	48.1	48.1
Percentage of egos in a relationship	60.0	82.9	76.4	66.0	68.6	52.0	71.0
Percentage of egos living in urban areas	63.8	46.2	58.6	62.6	72.8	48.1	61.7
Percentage of egos living alone	38.3	10.3	16.8	30.6	24.3	44.4	23.6
**Educational level**							
primary school	0.0	1.4	3.6	0.0	1.8	0.0	1.81
vocational high school	22.7	18.6	10.8	15.7	13.5	14.3	14.4
general or technical high school	47.7	38.6	44.3	44.9	43.6	38.1	43.5
higher vocational school	13.6	27.1	21.0	18.0	20.2	28.6	20.8
undergraduate	13.6	10.0	15.0	16.9	16.6	14.3	15.0
specialization	2.3	2.9	3.0	2.2	0.6	0.0	2.0
master’s degree	0.0	1.4	0.6	2.2	3.7	4.8	2.0
PhD	0.0	0.0	1.8	0.0	0.0	0.0	0.5

According to Wenger’s typology [24], we can approximately name the four obtained clusters as follows:

**CLUSTER 1—Wider community-focused support network** (*n* = 78). The respondents are very social with the highest number of alters. Support is given by family members, neighbors, and friends. Most are married and have completed more than high-school-level education. The social supporters come from the same household but also from other municipalities. Most of these respondents and social supporters live in a rural area.
**CLUSTER 2—Private restricted support network** (*n* = 181). The elderly people in this cluster have below-average social support, mostly provided by their partners or family members, and much less by their neighbors and friends. They have a low level of education and are generally men.
**CLUSTER 3—Less private restricted support network** (*n* = 99). They have above-average emotional and instrumental support, given not by their neighbors but by their partners, other family members, or friends. They are mostly women who live in an urban area.
**CLUSTER 4—Locally integrated support network** (*n* = 173). The respondents in this cluster are social ones. Support mostly comes from their friends, neighbors, and family members. Most of the alters live in the same municipality. Most of these respondents live in an urban area.

The respondents from the **cluster without social support** (Cluster 0, *n* = 47) are mostly men, living alone in an urban area with a high-school-level education. While the respondents from the **cluster without alters from the same municipality** (Cluster 5, *n* = 27) have a very small number of social supporters, they primarily live alone in a rural area.

The obtained typology is partially consistent with that proposed by Wegner [24]. Three out of the five types of social support networks proposed by Wenger [24] were found: (i) Locally integrated support network type; (ii) Wider community-focused support network type; and (ii) Private restricted support network type.

Only the Family dependent network type and Local self-contained network type were not found in the current study. The reason for not detecting the Family dependent network type is because the sample only includes a few respondents older than 80 years. Still, a new type of social network was detected: the Less private restricted support network type. It is similar to the Private restricted support network where the only supporter is the partner (this cluster contains an above-average share of men). The difference is that with the Less private restricted support network type the respondents obtain emotional and instrumental support not only from their partners, but also from their family members and friends (this cluster contains an above-average share of women from an urban area). It may therefore be concluded that among married couples female partners are the primary providers of social support to their husbands, while they (i.e., their wives) obtain social support from both their partners and also other supporters. Around 46% of all egos have one private restricted network type (Cluster 2 or Cluster 3), which is a bigger share of this network type than found in the results obtained by Wenger [24].

During the (first wave of the) the Covid-19 epidemic in Slovenia, 42% of the respondents enjoyed a rich social life with considerable social support (Wider community type and Locally integrated type). About 30% of the respondents are relying only on the support given by their partner—these are mostly men (Private restricted type). There are around 16% of respondents (Less private restricted support network type) who also receive emotional support from other family members or friends (apart from their partner).

Some elderly people are vulnerable since they lack informal social support, especially in a time like the coronavirus pandemic. We defined two types of such elderly people: those without any social support (about 8%) and those with the mentioned alters who were unable to come to the respondent due to the measure preventing people from leaving their own municipality (4.5%). These respondents have the lowest average number of listed alters.

## Discussion

Elderly people who are vulnerable in non-pandemic circumstances become even more so in times of a crisis such as the Covid-19 pandemic since the disease is especially dangerous for them [1, 2]. Studies show that social support plays an essential role in establishing and maintaining physical and mainly psychological health within the general population, particularly within the elderly population [39]. This population is very heterogeneous, engaged in different types of social support networks, not all of which are effective in their protecting role, meaning that some groups of the elderly are more vulnerable since they lack social support or the support is insufficient. The deficiency is not only limited to emotional support, but to other kinds of support like instrumental support. For example, during a ‘lockdown period’, some elderly people with chronic diseases might have limited access to medications, exercise, or physical therapy, which could worsen their health [13, 40, 41]. Reports also emerged that some elderly people and disabled had cancelled their home services to minimize physical contact out of fear of the virus [42].

Therefore, this study has aimed to identify different types of social support networks among the elderly population in Slovenia during the Covid-19 pandemic. The typology of different types of social support networks among the elderly referred to is that proposed by Wegner [24], who identified six different network types.

In the current study, the obtained typology of social support networks is generally consistent with that put forward by Wegner [24]. The only network type not present is the Family dependent network type, where elderly people are above 80 years of age. The absence of this network type might be because only those capable and willing to participate in web surveys were included, with the outcome that only a few people older than 80 years were included in the sample. Hence, no people from retirement homes and no one with a serious illness (that would have prevented them from completing the survey) were included in the study. Unfortunately, only the web survey approach was possible during the pandemic. However, part of the elderly population (e.g., those with serious illnesses) is also often not covered when other data collection modes are used.

Compared to Wenger’s study [24], a large share of elderly people is engaged with the Private restricted support network type (30%). In this network type, a couple (an older person with their partner, a child, or a relative) is somewhat isolated and helps each other. In the long term, these elderly people are vulnerable if their supporter dies or becomes unable to provide him/her with social support (e.g., due to illness or a conflict) [43]. In this case, they could shift over to the group that has no social support since elderly people have difficulties in finding new supporters.

A similar social support study was conducted 20 years ago in Slovenia with a similar sample size (*n* = 690). Based on that data, Hlebec [25] also performed a typology of social support networks with the egocentric network approach, but using a different clustering method. Our results differ from those obtained by Hlebec [22] for (at least) the following factors: (i) possible social changes in the 20-year period between the two studies; (ii) different data collection modes (telephone mode in 2000 vs web mode in 2020); and (iii) the Covid-19 pandemic. Despite these differences, the share of elderly people without any social support rose dramatically: in 2000, the number of elderly without any source with social support was 0.58% (4 elderly people), while in 2020 it was 7.8% (47 elderly people). As mentioned, these are mainly older single men. According to Chapman and Pancoast [44], older single men do not want to depend on others. However, as humans, they probably seek some kind of social support in places such as cafes and pubs. During the coronavirus epidemic in Slovenia, all of these places were closed and they therefore lost their social life and very probably felt miserable.

Besides those without any source of social support, people whose social supporters are all located in a different municipality might also experience deficient social support, primarily because of the sudden restriction on crossing over into a different municipality. The share of such elderly people is about 5%.

The characteristics of the groups of elderly people which are more vulnerable by lacking social support should be considered while designing appropriate interventions and combinations of care services for the elderly, not only during a pandemic like Covid-19 but in other circumstances like a natural disaster [45] (the key difference between other natural disasters and a pandemic is that other disasters bring community members together whereas a pandemic demands separateness [46]). Several studies [1, 14, 47] look at different measures that could be applied to protect the general population and vulnerable individuals during a future pandemic. Therefore, only some of them, relating to social support, are mentioned below.

The results of the current study stress the need for proactive and organized social support for those with limited informal social support since it is difficult for them to find appropriate sources of support, if they even exist. The difficulty might be, e.g., due to social stigma, lack of social competencies, or mobility issues (especially among those living in a rural area). The way this type of help is provided must respect cultural specifics and occur in a non-offensive way [42]. The role of non-governmental organizations as well as social workers is crucial in this regard [48].

Another possible measure is to increase digital literacy among the older population. Different digital technologies (such as online video communication services or online support groups) can serve as a space for prosocial behavior and empathy and can therefore help reduce feelings of loneliness and anxiety [46, 47]. Besides, tools for videoconferencing can help with socializing and providing emotional support (including professional emotional support, if available) while access to online communities and (governmental and non-governmental) websites assist by offering informational social support. The relevant informational social support during the Covid-19 pandemic must be spread by different types of media [48] because elderly people have varying access to different types of information technology (e.g., not only due to limited digital literacy but also, e.g., disabilities like blindness).

Other measures include promoting different coping approaches [46, 49] and approaches to minimize maladaptive responses, such as panic and paranoia regarding the disease and its transmission [14], and approaches to minimize stigma, discrimination, and ageism [50]. The latter are important since stigma, discrimination, and ageism can strongly influence the physical and emotional well-being of an elderly person as well as how their everyday needs are addressed [1, 23, 51].

This study did not cover elderly people incapable of participating in web surveys, such as those living in retirement homes. Retirement homes (which were insufficiently prepared for Covid-19) became hotspots for the coronavirus, not only in Slovenia but elsewhere [52–55]. To limit the spread of Covid-19, most retirement homes prohibited visits by relatives, even socializing within homes.

The study did not address specific psychosocial dimensions like general well-being, personality type, or perception of the adequacy of available social support. Such information could enable a better understanding of the underlying factors that lead to different types of social support networks. The mentioned psychosocial dimensions were not included in the questionnaire because measuring egocentric networks is cognitively demanding for respondents. Therefore, the study was limited to egos’ most essential personal characteristics. A follow-up study is planned to follow the most vulnerable elderly during the pandemic’s second wave by including relevant psychophysical characteristics of the respondents.

## Conclusion

The coronavirus pandemic has had a considerable impact on the physical and mental health of the general population, especially vulnerable populations like the elderly. Among the elderly population, some groups might be more vulnerable to experiencing feelings like loneliness, depression, and anxiety or, e.g., be unable to obtain their medications due to insufficient social support or because their social support is inadequate.

Therefore, this study aimed to identify different types of social support networks in the elderly population in Slovenia, estimate their prevalence, and discuss which types of social support networks might leave individuals susceptible during the coronavirus pandemic. The types of social support networks that were detected are generally consistent with Wenger’s typology [24]. One network type was not found due to the small number of respondents older than 80 years, while two additional network types were revealed. While over 50% of the respondents have satisfactory social support, a significant share exists with no source of social support and a relatively big share of those whose social supporters are all unavailable because they live in another municipality.

The results of the study hold relevance for designing appropriate interventions and combinations of care services for elderly people both during and after the Covid-19 pandemic.

## Supporting information

S1 Data The egocentric network data. The data are stored in the.sav format (Statistical Package for the Social Sciences by IBM). The data matrix contains personal data about the egos (e.g., gender, age) and data on their social support networks, as used for the clustering (e.g., the distribution of the distance between ego and his/her alters).
(SAV)

S1 Questionnaire Questionnaire in the Slovenian language. (PDF)

S2 Questionnaire Questionnaire in the English language. (PDF)
