# Supplementary material for: The social support networks of elderly people in Slovenia during the Covid-19 pandemic
Source: PLoS One. 2021 Mar 3;16(3):e0247993. doi: 10.1371/journal.pone.0247993 (PMC7928497; doi:10.1371/journal.pone.0247993)
Supplement: S1 Questionnaire — (PDF) [file pone.0247993.s002.pdf]

VPRAŠALNIK O OMREŽJIH SOCIALNE OPORE V ČASU PANDEMIJE KORONAVIRUSA

UVOD 1
V sodelovanju s Centrom za metodologijo in informatiko (Fakulteta za družbene vede), ponavljamo del raziskave izpred nekaj let – tokrat se osredotočamo na socialno oporo prebivalstva v času pandemije novega koronavirusa.

V nadaljevanju je vprašalnik, ki vam bo vzel manj kot 10 minut časa. Na osnovi vaših odgovorov bomo oblikovali predloge za izboljšanje socialne opore prebivalcev.

[gumb za nadaljevanje]

UVOD 2
Na počutje ljudi vplivajo tudi odnosi, ki jih imajo z ostalimi osebami. Z vprašanji, ki sledijo, vas želimo povprašati o osebah, ki so za vas pomembne, posebej sedaj, ko so neposredni stiki zelo omejeni. To so lahko partner, sorodniki, prijatelji, sodelavci, svetovalci, sosedje in ostali.

Pri vsakem vprašanju lahko navedete poljubno število oseb. Da bi zagotovili tajnost podatkov, lahko navedete le polna imena in začetnice njihovih priimkov in uporabljajte izbrano ime za posamezno osebo.

Kdo so osebe, s katerimi se družite v času omejitve gibanja? Bodisi v živo, bodisi po telefonu ali preko računalnika, tablice …
Da bi zagotovili tajnost podatkov, nam lahko poveste le polna imena in začetnice njihovih priimkov in uporabljajte izbrano ime za posamezno osebo. V spodnja okna zapišite imena, s katerimi imate navedene stike, ni potrebno izpolniti vseh oken.

[21 praznih okencev za vpis odgovora]

S kom se v teh dneh običajno pogovarjate o osebnih stvareh, ki so za vas pomembne?
Da bi zagotovili tajnost podatkov, nam lahko poveste le polna imena in začetnice njihovih priimkov in uporabljajte izbrano ime za posamezno osebo. V spodnja okna zapišite imena oseb, s katerimi imate navedene stike, ni potrebno izpolniti vseh oken.

[21 praznih okencev za vpis odgovora]

V času pandemije koronavirusa je priporočeno, da ne zapuščate stanovanja, da bi šli na primer po nakupih v trgovino ali po zdravila v lekarno. Na koga se v tem času obrnete za to vrsto pomoči?
Da bi zagotovili tajnost podatkov, nam lahko poveste le polna imena in začetnice njihovih priimkov in uporabljajte izbrano ime za posamezno osebo. V spodnja okna zapišite imena oseb, s katerimi imate navedene stike, ni potrebno izpolniti vseh oken.

[21 praznih okencev za vpis odgovora]

Spodnje vprašanje se pojavi respondentom, ki so navedli vsaj eno osebo.
Kako pogosto ste v stiku s to osebo osebno, po pošti, telefonu ali Internetu?

[za vsako navedeno osebo]

večkrat na dan	vsak dan	nekajkrat na teden	približno enkrat na teden	manjkrat		ne vem	ne želim odgovoriti

Spodnje vprašanje se pojavi respondentom, ki so navedli vsaj eno osebo.
Približno kako daleč od vas prebiva ta oseba?

[za vsako navedeno osebo]

stanuje v istem gospodinjstvu	stanuje v isti soseski	stanuje v istem kraju	stanuje v drugem kraju/občini	prebiva v tujini		ne vem	ne želim odgovoriti

Spodnje vprašanje se pojavi respondentom, ki so navedli vsaj eno osebo.
Kdaj ste se nazadnje "v živo" srečali z osebo, ki ste jo navedli:

[za vsako navedeno osebo]

vsak dan (živimo skupaj)	danes	včeraj	pred nekaj dnevi	pred enim tednom	pred nekaj tedni	pred enim mesecem	pred več kot enim mesecem

Spodnje vprašanje se pojavi respondentom, ki so navedli vsaj eno osebo.
V kakšni vrsti odnosa ste s to osebo? Če je vaš odnos s to osebo mogoče opisati na več načinov, lahko navedete več vrst odnosa.
Izberete lahko več odgovorov.

[za vsako navedeno osebo]

partner	otrok	vnuk	drug sorodnik	prijatelj	sosed	drugo	ne želim odgovoriti

Za konec sledi še nekaj vprašanj, ki se nanašajo na vas osebno.

Katerega spola ste?

moški
ženski
ne želim odgovoriti

Katerega leta ste rojeni?

[okence za vpis letnice rojstva]
ne želim odgovoriti

V kateri regiji živite?

Pomurska
Podravska
Koroška
Savinjska
Zasavska
Posavska
Dolenjska
Osrednjeslovenska
Gorenjska
Primorsko-notranjska
Goriška
Obalno-kraška
trenutno ne živim v Sloveniji
ne želim odgovoriti

V kakšni vrsti naselja živite?

urbano
ruralno
ne želim odgovoriti

Katera je vaša najvišja stopnja dosežene izobrazbe?

dokončana osnovna šola ali manj
srednja poklicna šola
splošna ali tehnična gimnazija
višja poklicna šola
dodiplomski študij
specializacija
magisterij
doktorat
ne želim odgovoriti

Koliko članov šteje vaše gospodinjstvo, vključno z vami?

1 član
2 člana
3 člani
4 člani
5 članov
6 članov
7 članov
8 članov ali več
ne želim odgovoriti

Kakšen je vaš zakonski stan?

samski / samska
poročen / poročena
zunajzakonska skupnost
ločen / ločena
poročen / poročena, a živim ločeno
vdovec, vdova
ne želim odgovoriti
