# Supplementary material for: The social support networks of elderly people in Slovenia during the Covid-19 pandemic
Source: PLoS One. 2021 Mar 3;16(3):e0247993. doi: 10.1371/journal.pone.0247993 (PMC7928497; doi:10.1371/journal.pone.0247993)
Supplement: S2 Questionnaire — (PDF) [file pone.0247993.s003.pdf]

QUESTIONNAIRE ON SOCIAL SUPPORT NETWORKS DURING THE CORONAVIRUS PANDEMIC

INTRODUCTION 1
With the Centre for Methodology and Informatics (Faculty of Social Sciences), we are repeating part of the research from a few years ago; this time, we are focusing on the social support of the population at the time of the new coronavirus pandemic.

Below is a questionnaire that will take you less than 10 minutes. Based on your answers, we will formulate proposals for improving the social support of the population.

[»next« button]

INTRODUCTION 2
People's wellbeing can be affected by the relationships they have with others. In the questions that follow, we want to ask you about the important people in your life, especially now that direct contacts are limited. These can be partners, relatives, friends, co-workers, counsellors, neighbors, and others.

You can list any number of people for each question. To ensure confidentiality, provide only first names and initials for their surnames.

Who are the people you have been socializing with during the time of social isolation? This can be face to face or by phone, computer, tablet, and so on.
To ensure the confidentiality of information, please only give us the full first names and initials for surnames; use each person's chosen name. Write the names in the windows below. It is not necessary to fill in all the windows.

[21 empty windows]

To whom do you usually talk to these days about personal things that are important to you?
To ensure the confidentiality of information, please only give us the full first names and initials for surnames; use each person's chosen name. Write the names in the windows below. It is not necessary to fill in all the windows.

[21 empty windows]

In the coronavirus crisis, it is advisable not to leave your residence, e.g., to go shopping or to the pharmacy. To whom do you turn for this type of help?
To ensure the confidentiality of information, please only give us the full first names and initials for surnames; use each person's chosen name. Write the names in the windows below. It is not necessary to fill in all the windows.

[21 empty windows]

The next question arises for respondents who named at least one person.
How often are you in contact with this person face to face or by mail, telephone, or the Internet?

[for each person]

Several times per day	Once per day	Several times per week	Once per week	Less than once per week		Do not know	Do not want to answer

The next question arises for respondents who named at least one person.
How far away does this person live from you?

[for each person]

Lives in the same household	Lives in the same neighborhood	Lives in the same city	Lives in another city/ municipality	Lives abroad		Do not know	Do not want to answer

The next question arises for respondents who named at least one person.
When was the last time you met in person with the person you listed?

[for each person]

We meet every day (we live together)	Today	Yesterday	Several days ago	One week ago	Several weeks ago	One month ago	More than one month ago

The next question arises for respondents who named at least one person.
What kind of relationship are you in with that person? If your relationship with this person can be described in several ways, you can list several types of relationships.
You can choose multiple answers.

[for each person]

Current or ex-partner	Father or mother (stepfather or stepmother)	Brother or sister (stepbrother or stepsister)	Child	Grandchild	Other relative	Co-worker or former co-worker
Current or former member of an organization	Current or former neighbor	Friend	Acquaintance	Current or former consultant	Other	Do not want to answer

Finally, here are a few more questions that apply to you personally.

Gender.

Male
Female
Do not want to answer

What year were you born?

[window for answer]
Do not want to answer

What region do you live in?

Mura
Drava
Carinthia
Savinja
Central Sava

Lower Sava
Southeast Slovenia
Central Slovenia
Upper Carniola
Littoral-Inner Carniola
Gorizia
Coastal-Karst
I do not currently live in Slovenia
Do not want to answer

What kind of location do you live in?

Urban environment
Rural environment
Do not want to answer

What is your highest level of education achieved?

Primary school
Vocational high school
General or technical high school
Higher vocational school
Undergraduate
Specialization
Master’s degree
PhD
Do not want to answer

How many members does your household have, including you?

1 member
2 members
3 members
4 members
5 members
6 members
7 members
8 members or more
Do not want to answer

What is your current marital status?

Single
Married
Extramarital community
Separated
Married, but live separately
Widower, widow
Do not want to answer
